# A microarray analysis of gnotobiotic mice indicating that microbial exposure during the neonatal period plays an essential role in immune system development

**DOI:** 10.1186/1471-2164-13-335

**Published:** 2012-07-23

**Authors:** Masahiro Yamamoto, Rui Yamaguchi, Kaori Munakata, Kiyoe Takashima, Mitsue Nishiyama, Kyoji Hioki, Yasuyuki Ohnishi, Masao Nagasaki, Seiya Imoto, Satoru Miyano, Atsushi Ishige, Kenji Watanabe

**Affiliations:** 1 Center for Kampo Medicine, Keio University School of Medicine, 35 Shinano-machi, Shinjuku-ku, Tokyo, 160-8582, Japan; 2 Tsumura Research Laboratories, Tsumura & Co., Ami, Ibaraki, 300-1192, Japan; 3 Human Genome Center, Institute of Medical Science, University of Tokyo, Tokyo, 108-8639, Japan; 4 Central Institute for Experimental Animals, Kawasaki, Kanagawa, 216-0001, Japan

**Keywords:** Hygiene hypothesis, Germ-free, Toll-like receptor, Type 1 interferon, MetaGene Profiler

## Abstract

**Background:**

Epidemiological studies have suggested that the encounter with commensal microorganisms during the neonatal period is essential for normal development of the host immune system. Basic research involving gnotobiotic mice has demonstrated that colonization at the age of 5 weeks is too late to reconstitute normal immune function. In this study, we examined the transcriptome profiles of the large intestine (LI), small intestine (SI), liver (LIV), and spleen (SPL) of 3 bacterial colonization models—specific pathogen**-**free mice (SPF), ex-germ-free mice with bacterial reconstitution at the time of delivery (0WexGF)**,** and ex-germ-free mice with bacterial reconstitution at 5 weeks of age (5WexGF)—and compared them with those of germ-free (GF) mice.

**Results:**

Hundreds of genes were affected in all tissues in each of the colonized models; however, a gene set enrichment analysis method, MetaGene Profiler (MGP)**,** demonstrated that the specific changes of Gene Ontology (GO) categories occurred predominantly in 0WexGF LI, SPF SI**,** and 5WexGF SPL, respectively. MGP analysis on signal pathways revealed prominent changes in toll-like receptor (TLR)- and type 1 interferon (IFN)-signaling in LI of 0WexGF and SPF mice, but not 5WexGF mice, while 5WexGF mice showed specific changes in chemokine signaling. RT-PCR analysis of TLR-related genes showed that the expression of interferon regulatory factor 3 (Irf3), a crucial rate-limiting transcription factor in the induction of type 1 IFN, prominently decreased in 0WexGF and SPF mice but not in 5WexGF and GF mice.

**Conclusion:**

The present study provides important new information regarding the molecular mechanisms of the so-called "hygiene hypothesis".

## Background

The so-called "hygiene hypothesis" suggests that reduced exposure of children to microbes is associated with increased prevalence of common allergies in developed countries [1-3]. At birth, the gastrointestinal tract is sterile and the neonatal immune response is characterized by a polarized T helper 2 (Th2) cytokine profile [4,5]. During gut colonization by commensal microorganisms, the gut immune system is constantly challenged by a myriad of bacterial and food antigens. Gut colonization apparently plays a major role in driving the initial Th2-skewed immune response toward a more finely balanced Th1/Th2 response, by boosting counterregulatory Th1 immune responses [6]. Numerous studies using animal models have suggested the possible involvement of immunoregulatory lymphocytes, e.g., regulatory T cells (Treg) and/or interleukin-10 (IL-10) producing B cells and cytokines (IL-10 and transforming growth factor-β) in intestinal homeostasis, which are driven by the intestinal bacterial burden [7-9]

Many epidemiological studies suggest that there is an inverse relationship between infections in early childhood and the subsequent development of allergic diseases [10,11]. Therapy with broad-spectrum antibiotics is frequently performed in pediatric practice and children receiving this therapy within their first year of life are particularly prone to develop allergic diseases later in life [12-14]. Studies in animal models, such as GF animals, have also suggested that microbiota play a critical role in normal development of the immune system [15]. Oyama et al. [16] reported that antibiotic use during infancy in mice promotes a shift in the Th1/Th2 balance toward Th2-dominant immunity. Further, they demonstrated that GF mice do not develop oral tolerance**,** which was restored by microbial reconstitution in neonatal (3**-**week**-**old) mice but not in older mice [17]. Impairment of immune tolerance has been shown to augment disease in various models of allergy and/or autoimmunity**,** including diabetes onset in NOD mice [18,19], collagen-induced arthritis [20,21]**,** and experimental colitis [22]. Taken together, these findings provide new perspectives on the pathogenesis and recurrence of these diseases [15,23,24]

In the present study, to elucidate the impact of microbes on the immune system during the neonatal period, we performed microarray analysis of LI, SI, LIV**,** and SPL of mice with or without enteric microbiota, and of GF mice reconstituted with microbiota at different ages.

In order to extract useful information from the massive amount of gene expression data obtained by microarray assay, we employed a gene set enrichment analysis approach in the present study. This type of analysis uses predetermined aggregations of genes (alternatively called gene sets, metagenes, gene modules**,** etc.) rather than individual genes to assess for coordinated expression in the samples. Single gene analysis may miss important effects on signaling because cellular processes often affect sets of genes acting in concert, with moderate effects on the strength of expression. Subtle but orchestrated changes of internally-related genes have often been found to be more important than a dramatic increase/decrease of a single gene. Therefore**,** it may be important to evaluate the statistical significance of changes in a gene aggregate or gene set, rather than in an individual gene. Since Subramanian et al.. [25] initially proposed “Gene Set Enrichment Analysis (GSEA)”, a number of algorithms optimized for this type of analysis have been developed [26-33]**,** and in the present study, we employed “MetaGene Profiler (MGP)” developed by Gupta et al. [34]. Unlike most existing methods, the main characteristic of MGP is that it evaluates statistical data for a set of genes independently from data of other gene sets. Because of the advantage of the analysis, it is logical to compare the results of tests for the same set of genes observed under different conditions, such as case–control experiments with multiple cases and time-course experiments, because the statistical evidence is evaluated using the same standard. MGP was therefore suitable for the present study**,** which required multiple cross comparisons of the same samples [35]. This study is part of a research project using GF and SPF mice of the IQI strain, which has been established as an inbred strain from ICR mice at the Central Institute for Experimental Animals (Kawasaki, Japan). We previously reported some of the results of microarray analyses conducted for the project. These studies indicated that 1) activation of the IFN-α system in LI differs significantly between GF and SPF mice [36], and 2) there is a striking commonality in transcriptome profiles between GF LI and SI, while the profiles of SPF LI and SI share almost nothing in common [37]. The present study, despite the use of different cohorts of animals, different versions of GeneChip arrays, and completely different strategies and algorithms for bioinformatics analysis from previous studies, has given support to our earlier findings. Furthermore, we demonstrated that the lack of neonatal encounter with commensal microorganisms may result in profound alteration of certain signaling pathways including TLRs, Rac 1**,** and type-1 IFN, which cannot be restored by later exposure to microbiota. These findings may provide important insights into the molecular events underlying the interaction between neonatal immune systems and commensal microbiota.

## Results

### Generation of gnotobiotic mice and targets of microarray analysis

**Figure 1:**
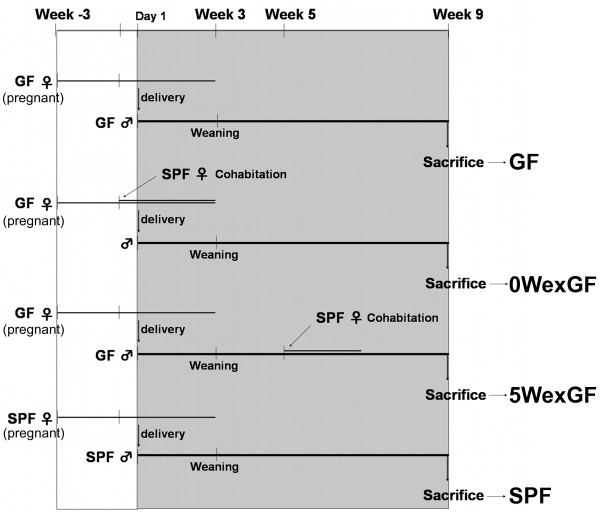
** Overview of the experimental procedure for bacterial reconstitution.** During the experiment, all mice (including SPF mice) were housed in a Trexler-type flexible film isolator in a standard germ**-**free state. For generation of 0WexGF (mice, i.e., GF mice with bacterial reconstitution at the time of delivery), pregnant GF mice were housed with SPF female mice 1 day before delivery and only male pups were retrieved after weaning. To generate 5WexGF mice (i.e., GF mice with bacterial reconstitution at 5 weeks old), since 5 weeks, GF male mice began to cohabit with SPF female mice of the same age. Male mice in all groups were sacrificed at 9 weeks of age.

In the present study we prepared 4 groups of mice with different status of enteric flora: GF, SPF, 0WexGF, and 5WexGF (Figure 1). Bacterial reconstitution was executed not by oral administration of fecal suspension but by cohabitation of pregnant GF mice with female SPF mice of the same age, because 1) contact with microorganisms via a wide variety of parenteral routes such as birth canal, skin, eyes, ears, nostrils, airway, urinary tract**,** and vagina are also critically important for the establishment of relevant microbial flora, which differ profoundly among these anatomical sites, and 2) it is impossible to administer bacterial suspension orally to neonatal mice immediately after delivery.

Using GF and SPF mice of the same strain, we have analyzed the gene expression profile**s** of LI and SI in previous studies [36,37]. In the present study, we have extended the target of transcriptome analyses to the SPL and LIV in addition to the intestines. SPL is a key part of body’s immune system and, therefore, comparison of the gene expression profiles of SPL and intestines may help to elucidate the cross-talk between intestinal local immunity and general immunity. The gene expression profile of LIV enzymes, especially those of steroid and xenobiotic metabolism, has been shown to be profoundly affected by intestinal microflora via nutrients and metabolites transported through portal vein [38]. Further, translocation of bacteria-derived substances, e.g., lipopolysaccharide to the LIV**,** is also known to have a great impact on the host immune system.

### GO analysis: overview and gene clustering

**Figure 2:**
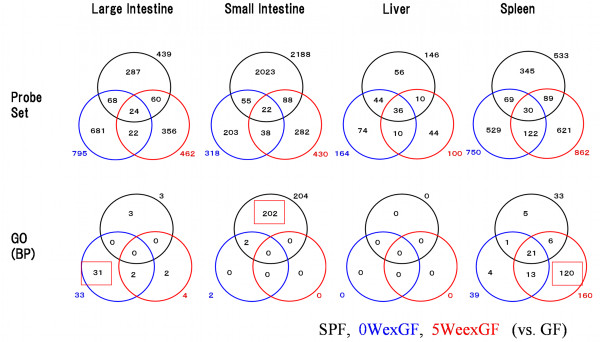
** Venn diagram showing differential expression of genes in the LI, SI, LIV, and SPL of 0WexGF vs. GF, 5WexGF vs. GF, and SPF vs. GF mice.** Upper row: The numbers of probe sets differentially expressed (p < 0.05). A total of 1498 probes sets were differentially expressed in the LI, 2711 in the SI, 274 in the LIV, and 1805 in the SPL. The greatest number of differentially-expressed probe sets (2188) was observed for SPF mouse SI. Only 22 ~ 36 probe sets were specifically co-regulated in all 3 comparisons in each tissue. Bottom row: The numbers of the GO BP categories having integrated p values of less than 0.05 are shown. In the LI, of the 38 overrepresented categories, 31 were specific for 0WexGF mice. In the SI, 202 of the 204 categories were specific for SPF mice. In the SPL, 160 of the 170 categories were specific for 5WexGF mice. No GO BP categories were overrepresented in the LIV.

Firstly, we have summarized the biological impact of enteric microbiota on host organs by MGP analysis on GO (http://www.geneontology.org/) categories. Figure 2 shows Venn diagrams of the numbers of differentially expressed genes and overrepresented GO categories of Biological Process (BP). Results from 3 comparisons (SPF vs. GF, 0WexGF vs. GF**,** and 5WexGF vs. GF) revealed alteration of an extremely large number of genes (probe sets) in the SI of SPF mice compared with those of 0WexGF and 5WexGF mice. There were approximately 22 to 36 commonly regulated probe sets in the LI, SI, LIV**,** and SPL among the 3 colonization models. At least 300 probe sets were listed in each group in the LI, SI, and SPL; however, no or only a small number of GO categories were overrepresented in most cases with the exception of LI in 0WexGF mice, SI in SPF mice, and SPL in 5WexGF mice. The overrepresented GO categories are listed in Table 1 (LI of 0WexGF), Table 2 (SI of SPF) and Table 3 (SPL of 5WexGF). Specific alteration of genes for antigen presentation in LI of 0exGF mice, and for energy cycle and nervous development in SI of SPF mice, were identified from these tables.

**Table 1:** Top 25 GO categories overrepresented in LI of 0WexGF mice compared with GF mice

**GOID**	**Term**	**Number of Probes**	**p_int**
GO:0042254	ribosome biogenesis and assembly	92	< 1.0E-18
GO:0019886	antigen processing and presentation of exogenous peptide antigen via MHC class II	14	6.18E-08
GO:0048511	rhythmic process	19	1.16E-07
GO:0006954	inflammatory response	48	6.79E-07
GO:0030300	regulation of cholesterol absorption	5	1.09E-06
GO:0006610	ribosomal protein import into nucleus	10	1.61E-06
GO:0042157	lipoprotein metabolic process	8	2.00E-06
GO:0001558	regulation of cell growth	64	2.63E-06
GO:0007166	cell surface receptor linked signal transduction	50	2.68E-06
GO:0050766	positive regulation of phagocytosis	10	3.64E-06
GO:0060048	cardiac muscle contraction	11	5.07E-06
GO:0007417	central nervous system development	17	6.65E-06
GO:0007413	axonal fasciculation	8	1.32E-05
GO:0006911	phagocytosis, engulfment	7	1.52E-05
GO:0006310	DNA recombination	27	1.54E-05
GO:0002504	antigen processing and presentation of peptide or polysaccharide antigen via MHC class II	6	2.46E-05
GO:0006950	response to stress	30	2.84E-05
GO:0000165	MAPKKK cascade	21	3.24E-05
GO:0007585	respiratory gaseous exchange	12	3.92E-05
GO:0006816	calcium ion transport	44	4.33E-05
GO:0042593	glucose homeostasis	18	5.19E-05
GO:0006414	translational elongation	14	5.69E-05
GO:0030901	midbrain development	8	6.54E-05
GO:0045582	positive regulation of T cell differentiation	6	7.88E-05
GO:0007229	integrin-mediated signaling pathway	36	8.14E-05

MGP analysis was performed on GO BP categories which contains 4 ~ 99 genes (“Number of Probes”). The categories were sorted according to the integrated p value (p_int) and the top 25 are represented. The categories related to antigen presentation are noted (e.g., “antigen processing and presentation of exogenous peptide antigen via MHC class II”, “inflammatory response”, “positive regulation of phagocytosis”, “phagocytosis, engulfment”, “antigen processing and presentation of peptide or polysaccharide antigen via MHC class II” and “positive regulation of T cell differentiation”).

**Figure 3:**
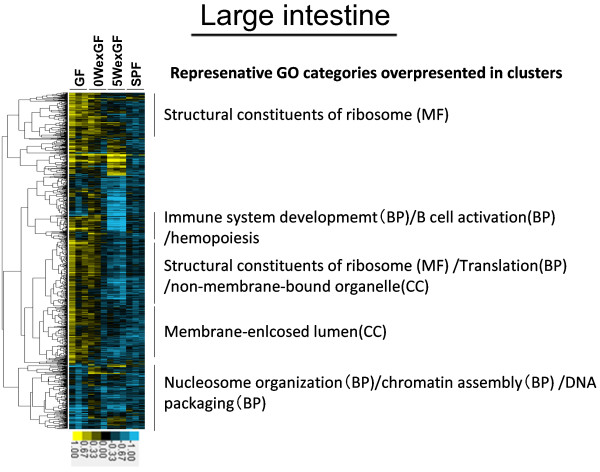
** Hierarchical cluster analysis of the 1498 differentially-expressed genes in the LI.** Expression was increased or decreased at a significance level of p < 0.05. Gene tree (Pearson correlation: left tree) shows correlated groups of genes and their expression patterns across all individual samples (top axis). The 5 groups highlight the separation of the gene clusters. MGP analysis was performed for each cluster and the top 1 ~ 3 GO categories (BP, MF, or/and CC) were listed. Colors show the range of expression from blue (decreased expression) to yellow (increased expression).

**Figure 4:**
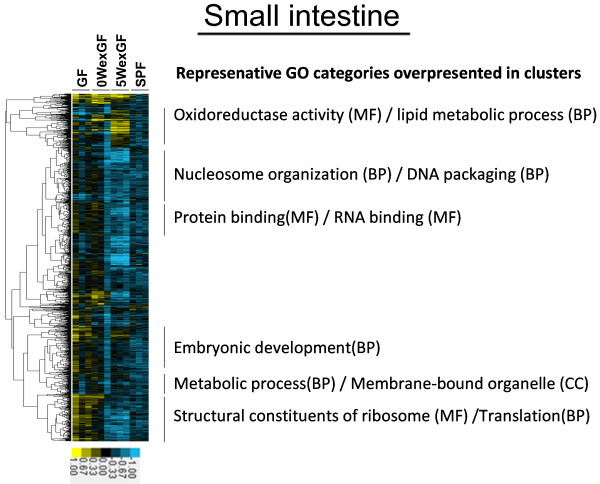
** Hierarchical cluster analysis of the 2711 differentially-expressed genes in the SI.** Expression was increased or decreased at a significance level of p < 0.05. Gene tree (Pearson correlation: left tree) shows correlated groups of genes and their expression patterns across all individual samples (top axis). The 6 groups highlight the separation of the gene clusters. MGP analysis was performed for each cluster and the top 1 ~ 3 GO categories (BP, MF, or/and CC) were listed. Colors show the range of expression from blue (decreased expression) to yellow (increased expression).

**Figure 5:**
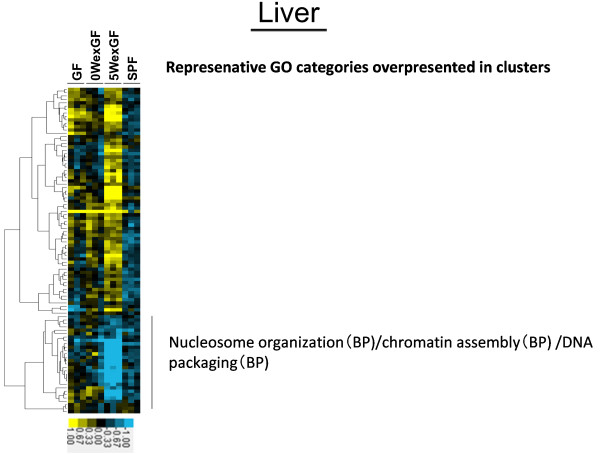
** Hierarchical cluster analysis of the 274 differentially expressed genes in the LIV.** Expression was increased or decreased at a significance level of p < 0.05. Gene tree (Pearson correlation: left tree) shows correlated groups of genes and their expression patterns across all individual samples (top axis). The 1 group highlights the separation of the gene clusters. MGP analysis was performed for each cluster and the top 1 ~ 3 GO categories (BP, MF, or/and CC) were listed. Colors show the range of expression from blue (decreased expression) to yellow (increased expression).

**Figure 6:**
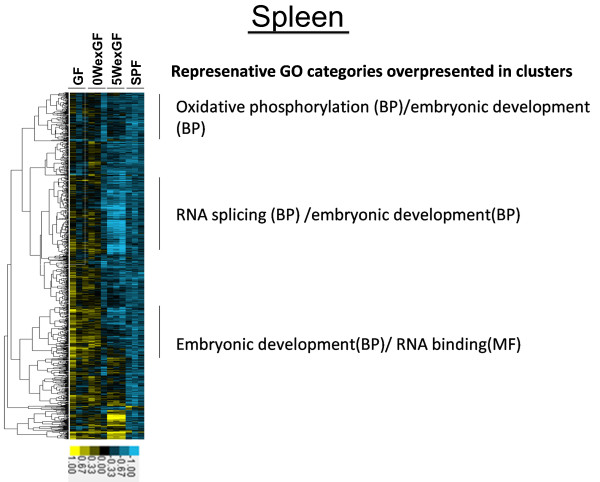
** Hierarchical cluster analysis of the 1805 differentially expressed genes in the SPL.** Expression was increased or decreased at a significance level of p < 0.05. Gene tree (Pearson correlation: left tree) shows correlated groups of genes and their expression patterns across all individual samples (bottom axis). The 3 groups highlight the separation of the gene clusters. MGP analysis was performed for each cluster and the top 1 ~ 3 GO categories (BP, MF, or/and CC) were listed. Colors show the range of expression from blue (decreased expression) to yellow (increased expression).

The results of unsupervised hierarchical cluster analysis of gene expression patterns are shown in Figure 3, Figure 4, Figure 5 and Figure 6. The clusters denoting overrepresented GO categories included immune system development/B cell activation/hematopoiesis (in LI) and metabolic process/lipid metabolic process (in SI).

### Analysis of signal pathway

**Figure 7:**
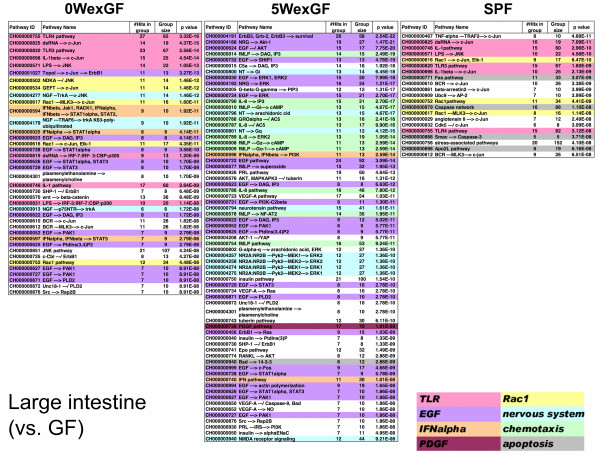
** Signal pathways overrepresented in LI of 0WexGF, 5WexGF and SPF mice compared to GF mice.** MGP analysis was performed using signal pathways and chains in the TRANTHPATH database. The pathways/chains were sorted according to the integrated p-value (q) and those with q < 0.001 have been represented. Some pathways/chains were classified by their biological contexts and colored as represented in the figure.

**Figure 8:**
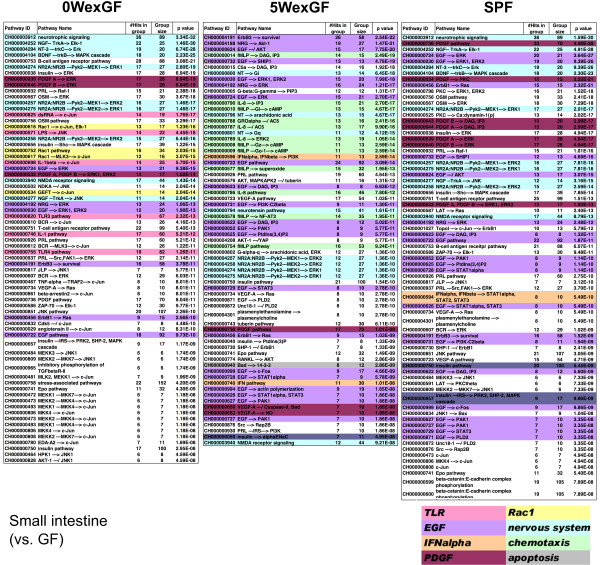
** Signal pathways overrepresented in SI of 0WexGF, 5WexGF and SPF mice compared with GF mice.** MGP analysis was performed using signal pathways and chains in the TRANTHPATH database. The pathways/chains were sorted according to the integrated p-value (q) and those with q < 0.001 have been represented. Some pathways/chains were classified by their biological contexts and colored as represented in the figure.

**Figure 9:**
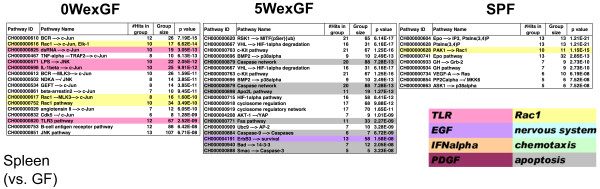
** Signal pathways overrepresented in SPL of 0WexGF, 5WexGF and SPF mice compared with GF mice.** MGP analysis was performed using signal pathways and chains in the TRANTHPATH database. The pathways/chains were sorted according to the integrated p-value (q) and those with q < 0.001 have been represented. Some pathways/chains were classified by their biological contexts and colored as represented in the figure.

Next, in order to gain information regarding the molecular events evoked by enteric microbiota, we applied the gene set enrichment analysis program MGP to the signal pathway database of TRANTHPATH. Figure 7, Figure 8, and Figure 9 show the signal pathways overrepresented in LI, SI and SPL. TLR, Rac1, and IFN-α signaling were commonly overrepresented in the LI of SPF and 0WexGF mice (note that “dsRNA/TLR3” pathways are closely interconnected with the pathways for IFN-α production) and epidermal growth factor (EGF) and nerve growth factor (NGF) signaling were common in the LI of 0WexGF and 5WexGF mice. There was low commonality between 5WexGF and SPF. In 5WexGF mice, specific alteration of gene expression for chemotaxis was indicated. The lists for SI differed from those of LI in SPF and 0WexGF mice (Figure 8). However, surprisingly, the list for SI seemed essentially the same as that of LI in 5WexGF mice (Figure 7 and Figure 8). In all 3 groups, the profiles of SI were characterized by large scale alteration of EGF, platelet-derived growth factor (PDGF) and NGF/neurotrophin signaling. Overrepresentation of TLR pathway and Rac1 signaling was observed in 0WexGF SI but not in SPF SI. In SPL, only a few overrepresented signal pathways passed the statistical criteria (q < 0.001), but overrepresentation of TLR pathway and Rac1 signaling in 0WexGF mice and apoptosis pathway signaling in 5WexGF mice was noted. No pathways reached statistical significance of q < 0.001 in LIV.

### Investigation of TLR signaling by quantitative RT-PCR

**Figure 10:**
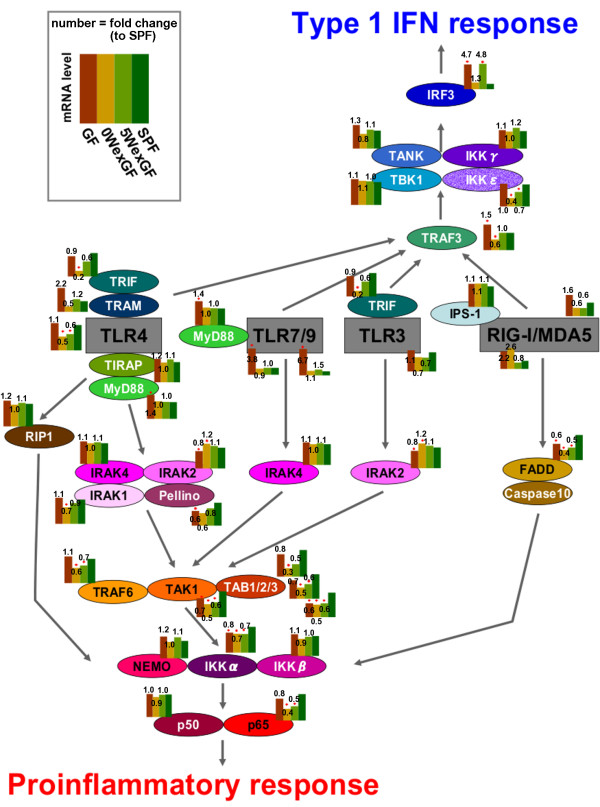
** Summary of the results of RT-PCR of TLR-related genes in LI.** The molecules involved in TLR signaling are shown in two clusters: one leads to the induction of proinflammatory cytokines in an NFκB-dependent manner, and the other leads to the production of type 1 IFN in an NFκB-independent manner. Expression levels are represented by columns indicating the relative value of each group normalized to the maximum value in the four groups. Number represents fold-change to the expression level in SPF. Brown, GF; ocher, 0WexGF; light green, 5WexGF; dark green, SPF. Differences compared with SPF mice were analyzed by Dunnet test. * p < 0.05.

Gene set enrichment analysis provides useful information about the statistical significance of changes in the expression of gene sets. However, the analysis does not provide information on the degree and direction of change of expression for any particular gene. Furthermore, it is unlikely that any existing database would include all of the signaling pathways involved in complex physiological and pathophysiological events. For examples, "TLR pathway" in the TRANTPATH database seemed to contain relatively little information on TLR7 and TLR9. Therefore, in order to obtain more information regarding differences in TLR signaling, we investigated the expression of various TLR-related genes by real time RT-PCR analysis in LI of the 4 groups of mice. The results are summarized in Figure 10 in terms of the difference of gene expression compared with SPF mice. The status of enteric microbiota (absence, presence, and introduction at different times) influenced various TLR-related genes in diverse ways. The most prominent and specific changes were seen in TLR7/9 (enteric microbiota, irrespective of colonization protocols, strongly decreased TLR7/9 expression) and IRF3 (expression was very low in SPF and 0WexGF mice and high in GF and 5WexGF mice).

## Discussion

In our previous study [36], using GF and SPF IQI mice and the old version of Affimetrix GeneChip (MG-U74Av2), we compared gene expression profiles of LI in GF and SPF mice. A list of genes most affected by the presence of enteric microbiota was generated by the simple Welch's t-statistic and enumeration in the order corresponding to the fold-change. The list clearly indicated that the expression of genes related to the induction and production of type 1 IFN, such as Irf3, Irf7, Isgf3g, Ifit1 and G1p2 (ISG15)**,** was markedly decreased in SPF mice compared with GF mice. In situ hybridization and immunohistochemistry indicated that these IFN-related genes were expressed mainly in lamina proprial CD11b^+^ cells. In another study [37], using a synthetic IFN inducer and an herbal medicine that enhances IFN production, we demonstrated that the difference in the steady-state expression level of these IFN-related genes was correlated with the difference in the timing of IFN-α release triggered by the inducer. In the present study, we used the same mouse strain but a different version of GeneChip (Mouse Expression 430A) and employed a completely different strategy and algorithm for bioinformatic analysis. In place of single gene analysis, we have adopted a gene set enrichment analysis with the primary aim of identifying the most affected gene sets in terms of statistical significance. The predetermined gene sets of categories, cascades and pathways have been furnished from public (GO) and commercial (TRANTHPATH) databases. The results indicated that, together with TLR signaling and Rac1 signaling, the signaling cascade for the production of type 1 IFN was most prominently affected in SPF and 0WexGF mice. Further, in good accordance with our previous paper [37], the changes in gene expression profiles were dissimilar between SI and LI of SPF mice**,** while those in GF mice showed substantial commonality between SI and LI.

The gene expression profile of SPF mice was similar to that of 0WexGF, particularly in LI, but not to that of 5WexGF mice. The main differences were observed in TLR, Rac1, and IFN-α signaling. TLRs play an important role in the recognition of microbes by host sentinel cells that contribute to subsequent innate and adaptive immune responses [39]. TLRs recognize molecular patterns specific for microbes to eliminate pathogens and engender commensal colonization of symbiotic bacteria [40]. Therefore**,** the difference in TLR signaling may contribute to the altered immune function of 5WexGF mice. Similarly, 5WexGF mice may have unique characteristics with respect to the IFN-α response, which is critically important not only for protection against infection by virus and certain microorganism, but also for control of autoimmune responses. Only in 5WexGF intestines, both SI and LI, large scale alteration of chemokine signaling was observed. In response to microbial stimuli, fetal intestinal epithelial cells release chemokines very rapidly, and this response may be a prerequisite for intestinal tolerance to commensal bacteria [41]. Acute chemokine release in response to microbial activation was subsequently maintained for several days in GF mice, but did not occur in mice harboring conventional microbiota due to tolerance acquisition at birth [42]. To clarify how the observed changes in the expression profile of chemokine signaling in 5WexGF mice relates to the failure to acquire immune tolerance, it may be necessary to examine chemokine production in epithelial cells isolated from 5WexGF intestines. Our findings collectively suggest that the encounter with environmental microbiota during the specific time interval within the neonatal period is critically important for the development of normal immune system responsiveness to microorganisms, both commensal and infectious.

Previous transcriptomics studies focusing on the role of commensal bacteria [43-46] differ from the present study with respect to animal species (mice vs. rats vs. piglets), tissues (epithelium/epithelial cells vs. whole intestines), ages at the time of sampling (pups vs. puberty vs. adult), means of manipulation of microbiota (conventionalization vs. monoassociation vs. antibiotic eradication of bacteria), microarray platforms**,** and statistical methodologies. However, the results of these studies are similar to ours in several respects such as prominent alteration of the expression profiles for antigen presentation, xenobiotic systems**,** and IFN signaling. To shed additional light on the "hygiene hypothesis", a careful comparison of our results with those of Schumann et al. [43] would be particularly valuable because in the latter study, enteric bacteria were eradicated by antibiotic treatment of neonatal rats from postnatal day 7 to 21. In the study by Schumann and colleagues, in addition to significant down-regulation of antigen presentation systems, expression of paneth cell products such as α-defensins, matrylsin, and type IIA phospholipase A2 decreased and expression of mast cell proteases increased as a result of a drastic reduction in enteric bacteria evoked by antibiotics. Similar changes were observed in the SI of GF mice compared with the other 3 colonization models in the present study (data not shown). Furthermore, Schumann et al.. have also found that eradication of enteric bacteria had a greater effect, in terms of the number of affected probe sets, in SI than in the colon. Clustering analysis of the affected probe sets in the study revealed only a few functional categories; i.e., endocytosis and vesicle-mediated transport in the proximal SI, immune response in the distal SI, and ion transport processes in the colon. The present analysis based on MGP identified expression changes for many functional categories, including proton transport and vesicle-mediated transport in the SI (Table 2) and immune responses in the LI (Table 1). In spite of the extensive differences in species, experimental settings, microarray platforms, and approaches to statistical analysis, a detailed comparative examination of these two transcriptome data sets might provide new insights into the impact of commensal microbiota colonization during the neonatal period.

**Table 2:** Top 25 GO categories overrepresented in SI of SPF mice compared to GF mice

**GOID**	**Term**	**Number of Probes**	**p_int**
GO:0015992	proton transport	67	< 1.0E-18
GO:0015986	ATP synthesis coupled proton transport	44	< 1.0E-18
GO:0006099	tricarboxylic acid cycle	39	< 1.0E-18
GO:0016192	vesicle-mediated transport	95	< 1.0E-18
GO:0006631	fatty acid metabolic process	63	< 1.0E-18
GO:0045454	cell redox homeostasis	46	1.11E-16
GO:0006754	ATP biosynthetic process	40	2.78E-15
GO:0006096	Glycolysis	56	5.32E-13
GO:0016481	negative regulation of transcription	68	1.05E-12
GO:0042254	ribosome biogenesis and assembly	96	2.27E-11
GO:0009987	cellular process	85	2.41E-11
GO:0001501	skeletal development	27	3.77E-11
GO:0006461	protein complex assembly	52	3.84E-11
GO:0021799	cerebral cortex radially oriented migration	5	5.23E-11
GO:0021813	cell-cell adhesion involved in neuronal-glial interactions involved in cerebral cortex glial-mediated radial cell migration	5	5.23E-11
GO:0021589	cerebellum structural organization	5	5.23E-11
GO:0021942	radial glia guided migration of Purkinje cell	5	5.23E-11
GO:0000160	two-component signal transduction system (phosphorelay)	31	6.78E-11
GO:0008654	phospholipid biosynthetic process	41	1.00E-10
GO:0007031	peroxisome organization and biogenesis	20	4.44E-10
GO:0007399	nervous system development	96	7.76E-10
GO:0044267	cellular protein metabolic process	26	1.06E-09
GO:0006396	RNA processing	75	1.15E-09
GO:0007162	negative regulation of cell adhesion	11	2.04E-09
GO:0007266	Rho protein signal transduction	38	2.27E-09
GO:0007229	integrin-mediated signaling pathway	36	8.14E-05

MGP analysis was performed on GO BP categories which contains 4 ~ 99 genes (“Number of Probes”). The categories were sorted according to the integrated p value (p_int) and top 25 are represented. The categories related to energy cycle are noted (e.g., “proton transport”, “tricarboxylic acid cycle”, “ATP biosynthetic process”, and “Glycolysis”).

## Conclusions

In summary, the gene expression profiles of mice with bacterial colonization at different times suggest that the encounter with environmental commensal microbiota during the specific time interval within neonatal period is essential for normal development of the immune system, especially of the LI. Microbiota-mediated development of regulatory circuits of TLRs and type I IFN seem to play a particularly important role. Rectification of chemokine expression might also be involved in microbiota-related immunological dysregulation because expression of chemokines was activated specifically in mice colonized at pre-pubertal ages. The present study provides important insights for clarification and refinement of the so-called "hygiene hypothesis".

**Table 3:** Top 25 GO categories overrepresented in SPL of 5WexGF mice compared with GF mice

**GOID**	**Term**	**Number of Probes**	**p_int**
GO:0007507	heart development	91	1.98E-13
GO:0006952	defense response	63	4.39E-12
GO:0016192	vesicle-mediated transport	76	3.47E-11
GO:0015992	proton transport	64	1.69E-09
GO:0001764	neuron migration	32	2.46E-09
GO:0007283	Spermatogenesis	94	2.65E-09
GO:0006816	calcium ion transport	41	3.52E-09
GO:0030036	actin cytoskeleton organization and biogenesis	52	3.81E-09
GO:0042127	regulation of cell proliferation	46	8.17E-09
GO:0016055	Wnt receptor signaling pathway	61	1.16E-08
GO:0007010	cytoskeleton organization and biogenesis	43	1.52E-08
GO:0016337	cell-cell adhesion	33	2.30E-08
GO:0018108	peptidyl-tyrosine phosphorylation	25	2.51E-08
GO:0008610	lipid biosynthetic process	76	2.69E-08
GO:0009987	cellular process	78	3.52E-08
GO:0006954	inflammatory response	48	4.44E-08
GO:0006874	cellular calcium ion homeostasis	23	6.93E-08
GO:0007166	cell surface receptor linked signal transduction	54	9.30E-08
GO:0005975	carbohydrate metabolic process	81	1.01E-07
GO:0016311	Dephosphorylation	61	1.76E-07
GO:0042981	regulation of apoptosis	83	2.21E-07
GO:0007420	brain development	30	3.64E-07
GO:0006935	Chemotaxis	45	4.40E-07
GO:0001701	in utero embryonic development	55	5.95E-07
GO:0009408	response to heat	26	8.93E-07

MGP analysis was performed on GO BP categories which contains 4 ~ 99 genes (“Number of Probes”). The categories were sorted according to the integrated p value (p_int) and top 25 are represented.

## Methods

### Animals

All animal experiments were performed in Central Institute of Experimental Animals (CIEA; Kanagawa, Japan). IQI/Jic mice were kept under SPF and GF mice were housed in a Trexler-type flexible film isolator in a standard germ**-**free state and screened on a weekly basis for germ-free status by sterile feces sampling and culturing on MRS-agar plates under aerobic and anaerobic conditions. Mice were housed in an air-conditioned room (temperature 24 ± 1 °C) with a controlled light/dark cycle (light on between 6:30 AM and 7:00 PM). Sterile food and water were available *ad libitum*. The mice were randomly divided into 4 groups; 10 male mice were included in each group. GF, SPF, 0WexGF, and 5WexGF mice (Figure 1). For generation of 0WexGF mice, pregnant GF mice were housed with SPF female mice 1 day before delivery and only male pups were retrieved after weaning. To generate 5WexGF mice, 5-week-old GF male mice were housed with SPF female mice. Mice in all groups were sacrificed at 9 weeks of age. All animal procedures were approved by the institution's ethical committee for care and use of laboratory animals in research.

### RNA extraction from mouse tissue

Mice were sacrificed and the LI, SI, LIV, and SPL were harvested for preparation of total RNA. Each frozen sample was homogenized in 1 ml/0.1 g tissue of TRI Reagent (Sigma-Aldrich Japan, Tokyo, Japan) with a Polytron tissue homogenizer (Kinematica, Littau-Lucerne, Switzerland) and incubated for 10 minutes at RT. Chloroform (0.2 ml/1 ml TRI Reagent) was added to the samples and the suspensions were centrifuged at 13,200 × *g* for 15 min at 4 °C. The water phase was transferred to a new tube and the RNA was prepared using a conventional isopropanol/ethanol precipitate technique.

### Microarray analysis

Total RNA extracted from mice (n = 3 per each group) were re-purified using RNeasy spin columns (Qiagen, Valencia, CA) according to the manufacturer's instructions. All samples were monitored using an Agilent Bioanalyzer (Agilent Biotechnologies, Boeblingen, Germany) and consistently demonstrated high-quality RNA (28 S/18 S ratio, ~2). The labeled cRNA prepared from 200 ng total RNA by *in vitro* transcription (Enzo Biochem, New York, NY) was fragmented, hybridized to a Mouse Expression 430 array (Affymetrix, Santa Clara, CA) using an Affymetrix fluidics station, and scanned with an Affymetrix scanner, according to the Affymetrix protocol. Data were uploaded to the Center for Information Biology Gene Expression Database (CIBEX, http://cibex.nig.ac.jp/index.jsp) and are available under accession ID CBX256. Data were analyzed using the Affymetrix Microarray Suite [37] v.5.0 with all of the parameters set at default values (a global normalization was applied). Probe sets that had 2 or 3 absent A MAS detection calls per group (3 samples) in all groups were excluded. Therefore, genes that had more than 2 present calls in any one of the groups were included in the analysis. Probe annotations were obtained from the Affymetrix NetAffx Analysis Center. Functional and signal pathway annotation of transcripts was based on Gene Ontology [47] and TRANSPATH [48] term assignments, respectively.

### Data clustering

Hierarchical clustering of subsets of genes was performed using clustering and analysis software (Cluster 3.0**;**http://bonsai.hgc.jp/~mdehoon/software/cluster/software.htm). The Pearson correlation coefficient (r) was chosen to compute distances between expression vectors (d = 1-r), and the complete linkage clustering algorithm was used to build the hierarchical tree.

### MetaGene Profiler (MGP)

MGP has been developed to evaluate the significance of predefined sets of genes from transcriptome data (http://metagp.ism.ac.jp/) [34]. The method, which employs a meta-analysis technique, accumulates statistical evidence from a set of genes in order to build a more powerful test than can be achieved by analyzing individual genes. In the present study, we first predefined the group of genes for each GO term: all 3 categories, i.e., BP, cellular component (CC), and molecular function (MF)**,** were used. More than 20,000 gene sets were annotated by GO terms. We also used gene sets defined by pathways in the TRANSPATH database. To obtain the p-values for individual genes, Welch’s *t* test was performed. The individual p-values of the genes included in the gene set were integrated to obtain the integrated p-value for the gene set, as described previously (http://metagp.ism.ac.jp/). A gene set containing too small a number of genes is, in principle**,** unsuitable for evaluation of overrepresented gene sets. Preliminary examination suggested that GO terms containing more than 100 genes provided relatively little information because these terms represent too broad a concept to give a foothold for further biological investigation. Therefore MGP analysis was applied to gene sets consisting of 4–99 genes.

### Quantitative real time RT-PCR

For RT-PCR, total RNA was extracted as described above (n = 6 per group). The cDNA samples were synthesized using an Improm-IITM Reverse Transcriptase kit (Promega Corporation, Madison, Wl) according to the manufacturer’s instructions. Briefly, 5 μl of RNA (50 ng) and primer were added to 15 μl of reverse transcription reaction mix (Improm-IITM Reverse Transcriptase system). Annealing was performed by placing the tubes in a controlled-temperature heat block equilibrated at 25 °C and incubated for 5 minutes. Extension was performed in a controlled-temperature heat block at 42 °C for up to 1 hour. The extension temperature was optimized between 37 °C and 55 °C. Real time RT-PCR was performed using the TaqMan® Gold RT-PCR Kit (Applied Biosystems, Foster City, CA) according to the manufacturer’s instructions. An ABI Prism 7900HT (Applied Biosystems) was used with the following thermal cycling conditions: 1 cycle at 50 °C for 2 min, 1 cycle at 95 °C for 10 min, 40 cycles each at 95 °C for 15 sec and 60 °C for 1 min. Data were normalized against Irf1 [36,37].

### Statistical analyses

PCR data were calculated as the mean values and are represented in Figure 10 by columns indicating the relative value of each group**,** which was normalized to the maximum value in the 4 groups. Differences in mean values among groups were analyzed by Dunnet test and were considered significant at p < 0.05. The statistical methods used for the microarray analysis are described above.

## Abbreviations

0WexGF, Germ-free mice with bacterial reconstitution at the time of delivery; 5WexGF, Germ-free mice with bacterial reconstitution at 5 weeks of age; BP, Biological Process; CC, Cellular Component; GF, Germ-free; GO, Gene Ontology; GSEA, Gene set enrichment analysis; IFN, Interferon; IL, Interleukin; LI, Large intestine; MF, Molecular Function; MGP, MetaGene Profiler; SI, Small intestine; SPF, Specific pathogen free; Th, T helper; TLR, Toll like receptor; Treg, Regulatory T.

## Competing interests

The authors declare that they have no competing interests.

## Authors’ contributions

MY participated in the design of the study, data analysis, coordination, and drafted the manuscript. RY carried out microarray data analysis including MGP. KM participated in animal experiments. MiN and KT carried out RT-PCR. AI, KH and YO participated in the design and coordination of the microflora reconstitution experiments. MaN, SI, and SM participated in the design of statistical and bioinformatics analyses. KW conceived the study and participated in its design and coordination. All authors read and approved the final manuscript.
